# Acute Submandibular Sialadenitis

**DOI:** 10.7759/cureus.24435

**Published:** 2022-04-24

**Authors:** Megan Vu, Carlos Lopez Ortiz, Marcos Sosa, Bryna Peplinski, Latha Ganti

**Affiliations:** 1 Emergency Medicine, Trinity Preparatory School, Winter Park, USA; 2 Emergency Medicine, HCA Florida Ocala Hospital, Ocala, USA; 3 Emergency Medicine, University of Central Florida, Orlando, USA; 4 Emergency Medicine, Envision Physician Services, Plantation, USA; 5 Obstetrics and Gynecology, Lakeland Regional Health Medical Center, Lakeland, USA; 6 Emergency Medicine, University of Central Florida College of Medicine, Orlando, USA

**Keywords:** emergency department, submandibular sialadenitis, sialogogue, salivary gland stone, sialadenitis

## Abstract

Sialadenitis is a stone in the salivary system that often presents as acute pain and swelling and can cause significant distress to patients. Here, we present a case of submandibular sialadenitis in a 41-year-old woman who experienced sudden-onset facial and neck pain and swelling. In addition, we discuss the diagnosis and emergency department management.

## Introduction

Submandibular sialadenitis (SS) is the inflammation of the submandibular gland, which is frequently triggered by salivary stasis that leads to the production of bacteria at the back of the mouth. The glands are located in the submandibular triangle, which is covered by a layer of deep cervical fascia. The mylohyoid muscle separates the superficial and deep lobes of the glands. The submandibular glands drain into the mouth via Wharton’s duct, which is between the sublingual gland and hyoglossus muscle. On the floor of the mouth, the submandibular glands open through a small opening lateral to the frenulum on the floor of the mouth [1].

The parotid gland is the largest of the salivary glands. Located superficially, it is covered by a superficial layer of the deep cervical fascia, forming the parotid space. The parotid space is made up of the facial nerve, auriculotemporal branches of the mandibular division of the trigeminal nerve, intraparotid lymph nodes, the external carotid artery, and the retromandibular vein [2].

The submandibular gland is the second largest of the salivary glands; it is located at the angle of the mandible and the submandibular and sublingual spaces. The submandibular duct rises up from the anterior border of the submandibular gland and goes through the sublingual space between the mylohyoid muscle/sublingual gland and the hyoglossus/genioglossus muscles [2].

Patients afflicted by SS may have a swollen submandibular gland, pain, and a foul taste in the mouth or xerostomia [3]. The exact prevalence of SS is not clear. It accounts for 10% of all cases of sialadenitis and about 0.001-0.002% of all hospital admissions. While it commonly affects older, dehydrated patients, it does not have a preferred age or sex range. Typical causes of SS include bacteria (*Staphylococcal aureus*, *Haemophilus influenzae*, Gram-negative aerobes, anaerobes), viruses (mumps, human immunodeficiency virus), actinomyces, tuberculosis, obstructive issues (sialolithiasis, ductal structure, ductal foreign body, external compression of duct), inflammatory causes (post-radiation sialadenitis, contrast-induced sialadenitis, radioiodine treatment), drug-induced, autoimmune sialadenitis, and, finally, granulomatous sialadenitis (sarcoidosis, xanthogranulomatous sialadenitis) [1].

Major risk factors of SS include reduced salivary secretion, duct obstruction, old age, poor oral hygiene, postoperative state, intubation, use of anticholinergic agents, and microbes. Typical treatment or management techniques of SS include hydration, warm compresses, massage, pain relief with analgesics, sialogogues, and oral hygiene [1].

## Case presentation

**Figure 1:**
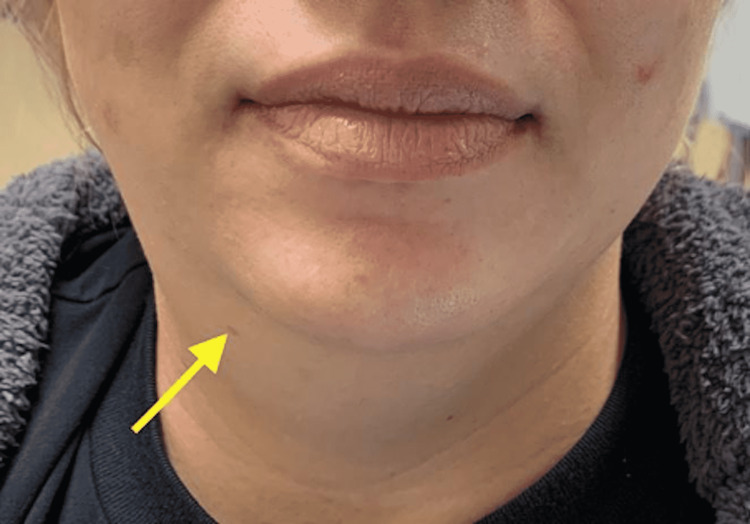
Clinical photograph of the patient with submandibular swelling.

A 41-year-old female presented to the emergency room with a sudden onset of right-sided neck swelling that started a few hours prior after drinking a milkshake. She denied any frank pain unless she was trying to swallow. The acute onset of the swelling was quite alarming to her. She had never experienced pain while swallowing. The pain during swallowing was not in her throat or her esophagus, but rather on the right side of her neck where the swelling was. She initially thought she had been stung by something, but this was not the case. Furthermore, the site of swelling was not itchy or red, or warm to the touch. She denied exposure to any new medications or foods. She had no history of allergic reactions to food. She had previously consumed the same milkshake too. She denied any fever, chills, chest pain, shortness of breath, nausea, vomiting, diarrhea, abdominal pain, headache, or rashes. She did not take any medications prior to arrival. Her vital signs were temperature of 97.6°F, blood pressure of 132/81 mmHg, respiratory rate of 18 breaths per minute, pulse of 77 beats per minute, and oxygen saturation of 97% on room air. Physical examination was remarkable for visible swelling of the right submandibular area (Figure 1).

Otherwise, the patient was well-appearing, albeit slightly anxious. Examination of the pharynx was normal, without erythema or edema. Lungs were clear to auscultation bilaterally, and there were no signs of urticaria. Laboratory analysis was remarkable for leukocytosis (Table 1).

**Table 1:** Patient’s laboratory values.

Laboratory test	Reference range	Test result
Chemistry		
Sodium	136–145 mmol/L	137
Potassium	3.5–5.1 mmol/L	4.0
Chloride	98–107 mmol/L	101
Carbon dioxide	21–32 mmol/L	30
Blood urea nitrogen	7–18 mg/dL	17
Creatinine	0.6–1.3 mg/dL	0.69
Estimated glomerular filtration rate	>90 mL/minute	>60
Blood urea nitrogen/Creatinine ratio		25
Glucose	74–110 mg/dL	106
Calcium	8.5–10.1 mg/dL	9.3
Total bilirubin	0.2–1.0 mg/dL	0.2
Aspartate aminotransferase	15–37 U/L	16
Alanine aminotransferase	12–78 U/L	19
Total alkaline phosphatase	46–117 U/L	97
Total protein	6.4–8.2 g/dL	7.4
Albumin	3.4–5.0 g/dL	3.8
Serum human chorionic gonadotropin, Qual	Negative	Negative
Hematology		
White blood cell count	4.0–10.5 ×10^3^/µL	11.5
Red blood cell count	3.93–5.22 ×10^6^/µL	4.89
Hemoglobin	11.2–15.7 g/dL	11.4
Hematocrit	34.1–44.9%	37.7
Platelet count	150–400 ×10^3^/µL	439

**Figure 2:**
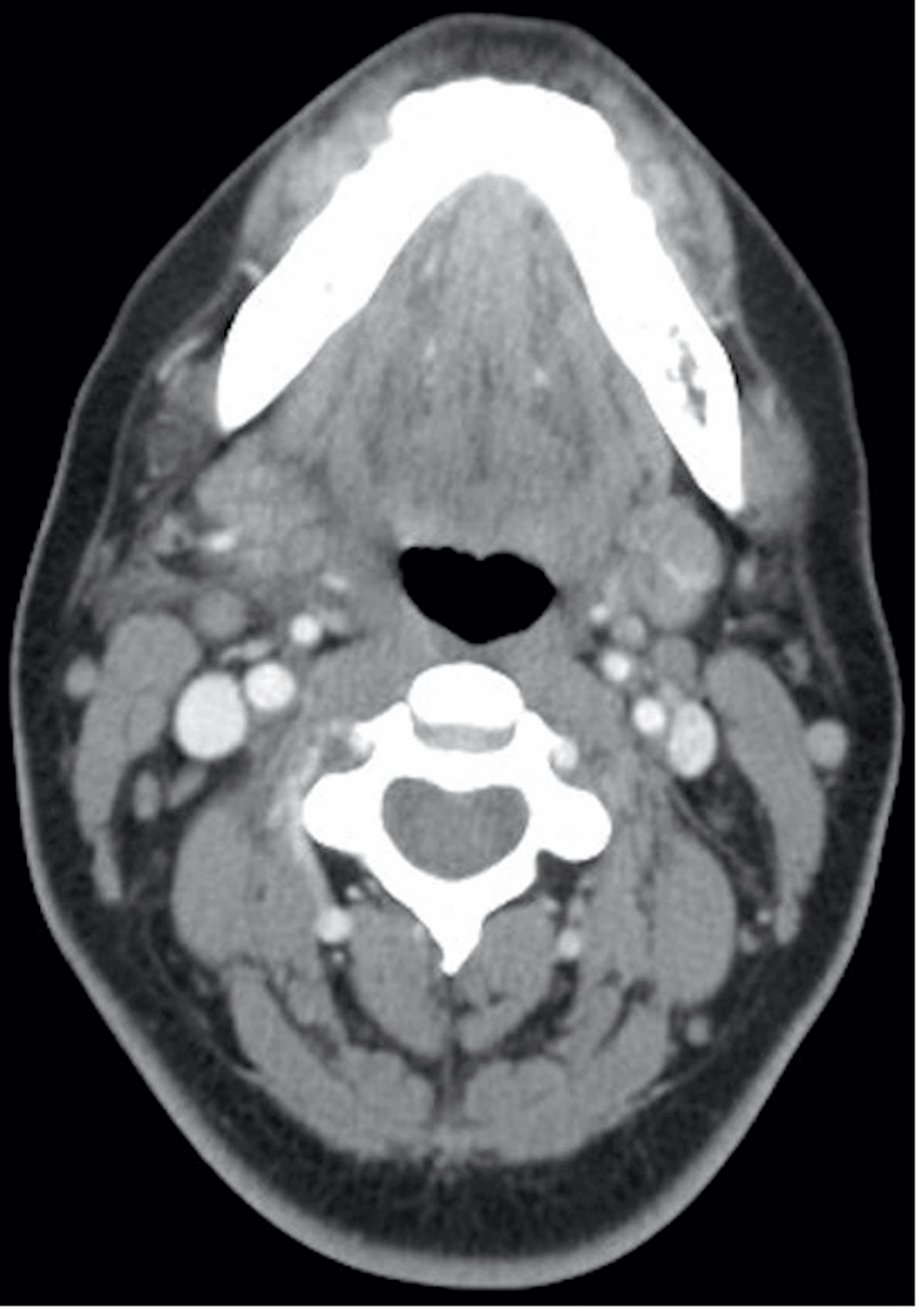
Computed tomography scan demonstrating submandibular sialadenitis.

The patient was given 30 mg of ketorolac and 1 g of ceftriaxone intravenously. Contrast computed tomography (CT) scan of the neck demonstrated asymmetric enlargement of the right submandibular gland with evidence of edema within the gland and the surrounding inflammation, consistent with right SS (Figure 2).

## Discussion

**Figure 3:**
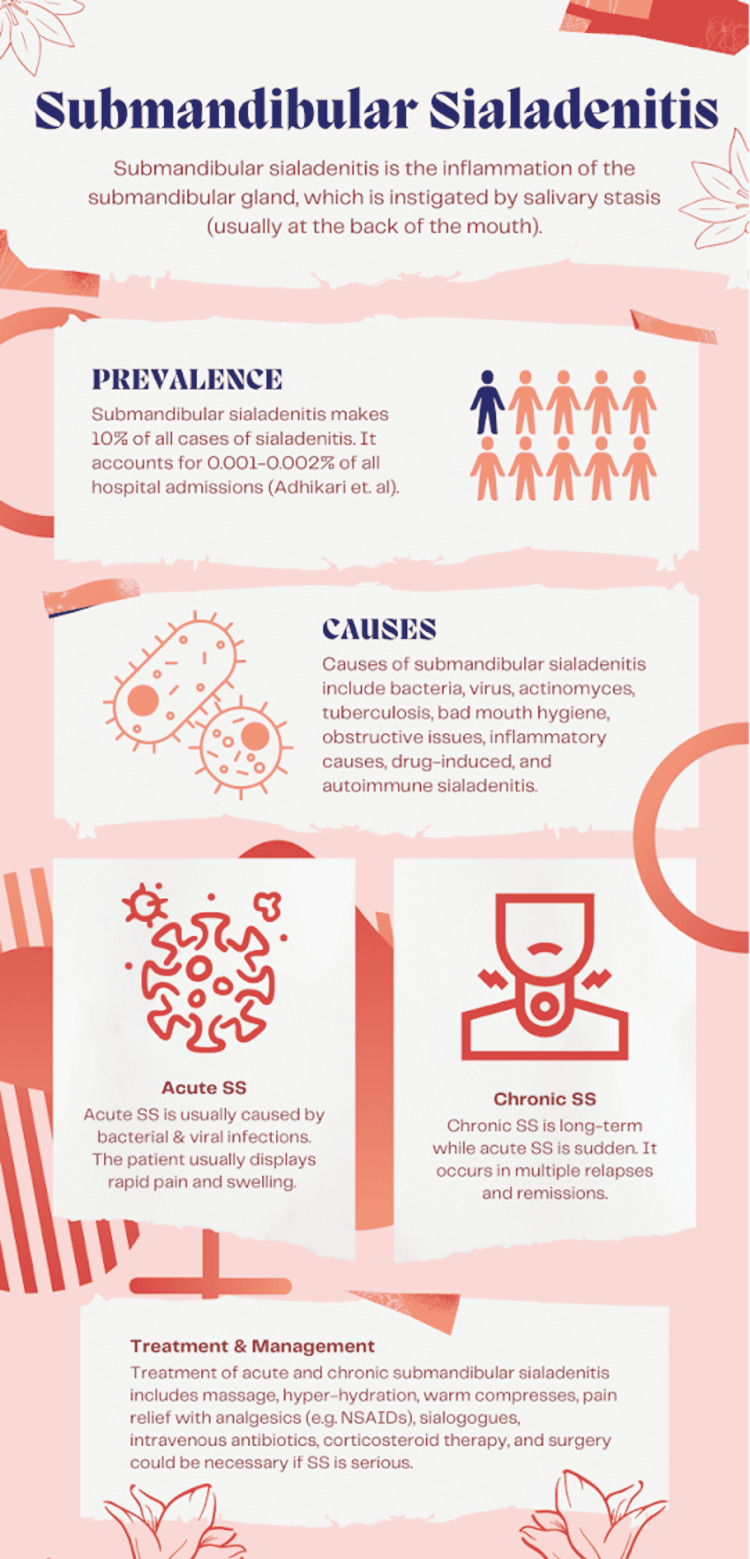
Submandibular sialadenitis.

Overall, this case study represents an example of the non-discriminatory nature of SS. It has the potential to happen to anyone regardless of age and sex (Figure 3).

SS is usually caused by salivary strictures (stenosis), which is when a salivary duct is narrowed. It develops in certain circumstances such as when a patient undergoes radioactive iodine therapy for thyroid cancer. Iodine is prominent and secreted within the saliva, mostly from the sodium iodide symporter in the basement membrane lining the intralobular ducts [4]. Other causes include autoimmune diseases (such as Sjögren syndrome), infectious diseases, previous radiotherapy, and amyloidosis. SS can also be caused by acute or chronic infective, obstructive, immunoglobulin G4-related sialadenitis (IgG4-RS), lymphoepithelial, granulomatous, and post-treatment sialadenitis [3].

There are typically three types of SS: acute, chronic, or acute on chronic [3]. As such, there are different treatments for each variation. Acute SS is caused by salivary stasis in seriously dehydrated patients (i.e., terminally ill, postoperative, neonates), with *Staphylococcus aureus* as the most common pathogen. Patients have painful parotid swelling and discharge from the duct. Imaging shows that acute SS has an enlarged right parotid gland with enhanced abscess and hyperintense fluid [3]. Usually, acute SS is expected to resolve in a week, but edema may take longer to disappear [1]. Chronic SS is caused by episodes of acute inflammation following glandular destruction. Imaging shows that chronic SS of the parotid glands has a granular appearance, contains small cysts, and a mild inhomogeneous pattern of enhancement of both parotid glands [3,5,6]. It could also be induced by iodine-131 therapy; the most common manifestation of I-131-associated sialadenitis was severe stenosis within the distal salivary duct [5]. The prognosis of chronic SS is not as good as acute SS because it can have multiple relapses and remissions [1].

Risk factors of SS include recurrence, abscess formation, and dental decay. Abscess formation can lead to the infection spreading across the fascial planes of the neck. Hypofunction of the salivary gland can make it produce less saliva, thus leading to decreased protection from acid erosion, which would promote dental decay. Salivary hypofunction in elderly patients can occur due to polypharmacy causing dehydration and xerostomia leading some to consider the elderly an “at-risk” population for sialadenitis [7].

There are different treatments for each variation of SS. Acute SS requires conservative treatment, massage, hyper-hydration, warm compresses, and pain relief with analgesics such as non-steroidal anti-inflammatory drugs and sialogogues. Intravenous antibiotics could be necessary in certain circumstances. Corticosteroid therapy is also an alternative if swelling is noteworthy and there is no contraindication. On the other hand, chronic sialadenitis is treated differently. Indeed, hydration, oral hygiene, pain relief, and sialogogues are used for chronic SS as well. Broad-spectrum antibiotics may be necessary if there is an infection. If a salivary gland stone must be removed, interventional sialendoscopy, direct surgical removal, extracorporeal shock wave lithotripsy under ultrasonic guidance, and excision of the salivary gland are options [1]. However, up to 50% of the cases do not respond to these treatments [2].

SS is commonly diagnosed through conventional sialography, magnetic resonance imaging sialography, ultrasonography, or plain radiography [2,3]. A study of 31 patients designed to quantitatively assess normal submandibular glands and SS by utilizing CT demonstrated that CT was also an alternative means to diagnose SS [8]. However, sialendoscopy is the most popular method of diagnosis because it provides a vivid depiction of the ductal system [2].

As such, sialendoscopy is suggested to be part of the management of SS [2,6]. For example, one study reported a 75-100% improvement rate after including sialendoscopy in the treatment of 221 patients [9]. Sialendoscopy is also a highly effective treatment as it preserves the submandibular glands and their functions with a success rate of 80-100% [2]. A study of 115 patients who received radioiodine therapy after total thyroidectomy concluded that SS is mostly managed by conservative treatment. Interventional sialendoscopy is a good option for partial ductal stenosis [10].

## Conclusions

This case demonstrates the prevalence and the non-discriminatory nature of SS among all ages and sexes. Treatment of SS depends on the type of SS (i.e., acute or chronic) and its underlying cause.
